# Countrywide Survey of Plants Used for Liver Disease Management by Traditional Healers in Burkina Faso

**DOI:** 10.3389/fphar.2020.563751

**Published:** 2020-11-30

**Authors:** André Tibiri, Sébastien Boria, Tata Kadiatou Traoré, Noufou Ouédraogo, Aude Nikièma, Souleymane Ganaba, Jean-Marie Compaoré, Issiaka Ouédraogo, Innocent Pierre Guissou, Maëlle Carraz

**Affiliations:** ^1^ Laboratoire De Biologie Appliquée, Département De Médecine Et Pharmacopée Traditionnelles-Pharmacie (MEPHATRA-PH), Institut De Recherche En Science De La Santé, CNRST, Ouagadougou, Burkina Faso; ^2^ Airbus Defense and Space, Toulouse, France; ^3^ Laboratoire De Développement Du Médicament, Université De Ouagadougou, Ouagadougou, Burkina Faso; ^4^ UMI CNRS 3189, Institut Des Sciences Des Sociétés, CNRST Ouagadougou, Ouagadougou, Burkina Faso; ^5^ Département Environnement Et Forêts, Institut De L’Environnement Et De Recherches Agricoles, Ouagadougou, Burkina Faso; ^6^ Fédération Nationale Des Tradipraticiens De Santé Du Burkina (FENATRAB), Ministère De La Santé Du Burkina Faso, Ouagadougou, Burkina Faso; ^7^ Service De Gastro-Entérologie, Centre Hospitalier Universitaire Yalgado Ouédraogo (CHU-YO), Ouagadougou, Burkina Faso; ^8^ UMR 152 Pharma-Dev, Université De Toulouse, IRD, UPS, Toulouse, France

**Keywords:** ethnopharmacology, Burkina Faso, liver disease, hepatitis, hepatocarcinoma

## Abstract

Liver disease is highly prevalent in Africa, especially in the western African country Burkina Faso, due to the presence of multiple biological and chemical aggressors of the liver. Furthermore, diagnosis and appropriate care for liver disease are uneven and usually insufficient. This drives local communities to turn to folk medicine based on medicinal plants from healers. Small scale, ethnopharmacological studies on reputed hepatoprotective plants have been carried out in defined regions worldwide, but so far, no study has been carried out on a countrywide scale. Therefore, we have explored traditional healers’ practices in all thirteen regions of Burkina Faso. We interviewed 575 healers and we compiled a database with 2,006 plant entries. Here, we report results on liver nosology, liver pathologies, medicinal plants used for liver disease, and traditional practices through the lens of Burkinabe healers. Our goal was to give a full inventory of medicinal plants used to treat liver disease and to determine if there was consensus on the use of specific plants for specific symptoms. Analysis of the medicinal plants in use across the whole country provides local communities with a wider evidence base to determine which plants may be more effective in treating liver disease and could provide the scientific community, with a shortlist of plants suitable for chemical and pharmacological investigation to validate the plants’ therapeutic role.

## Introduction

Burkina Faso (formerly Upper Volta) is a landlocked west African country of 274,200 square kilometers that shares borders with six nations: Mali to the north and west, Niger to the east, Benin, Togo, Ghana, and Ivory Coast to the south. It has a homogenous landscape and is relatively flat, the highest point does not exceed an elevation of 750 m. Its climate is tropical with a rainy and a dry season and the country includes three main agro-climatic zones: north (the Sahelian part), middle (the northern Sudanian part), and south (the southern Sudanian part with forests and fruit trees). The middle and south zones contain the main vegetation of the country (Thiombiano and Kampmann, 2010). In the southern, Sudanian area of woody savannah, there are protected species such as *Vitellaria paradoxa* C. F. Gaertn (Sapotaceae), *Parkia biglobosa* (Jacq.) G. Don (Leguminosae), and *Lannea acida* A. Rich (Anacardiaceae), while in the forests, there are protected species such as *Isoberlinia doka* Craib & Stapf and *Isoberlinia tomentosa* (Harms) Craib & Stapf (Leguminosae). By contrast, the northern Sahelian part is rich in thorny trees such as Leguminosae (*Mimosa* genus) and Zygophyllaceae (*Balanites* genus) families (Bognounou et al., 2006). The country consists of 13 main regions, subdivided into 45 provinces, and is essentially rural with approximatively 80% of the inhabitants employed in agricultural and pastoral activities. In 2016, the population was 19.5 million, with a large number of the population concentrated in the center and belonging mainly to the *Mossi* socio-cultural group. The country has high cultural and religious diversity. The three main religions are Islam (60.5%), Christianity (23.2%), and Animism (15.3%), and there are more than 60 different socio-cultural groups. To the west, the cultural diversity is larger with the presence of groups such as the *Mossi*, *Bobo*, *Bwaba*, and *Samo* among others. The *Gulmantché* and *Peuls* groups are found in the east and north, respectively.

Globally, liver cancer (hepatocarcinoma) has the fourth highest age standardized cancer mortality rate according to the 2018 GLOBOCAN data (http://www.gco.iarc.fr). Epidemiological studies have estimated that 3.5% of the global population are chronically infected by viral hepatitis B (HBV) and the prevalence of HBV in children in Africa is also 3%. Moreover, we know that only a small proportion of people with viral hepatitis have been diagnosed, and among these patients, treatment has reached only a small fraction. Consequently, chronic viral hepatitis is a major health issue worldwide but particularly in Africa and Asia. Chronic viral hepatitis B and C causes liver fibrosis and subsequently cirrhosis and hepatocarcinoma (Petrizzo et al., 2018). In Burkina Faso, hepatocarcinoma also has the fourth highest cancer mortality rate in persons after cervical, prostate, and breast cancers. Liver cancer is the second most common cause of cancer death in persons (1,269 deaths in 2018) and the most common cause of cancer death in males. In Burkina Faso, liver disease accounted for 3,105 deaths in 2017 (2.3% of total deaths) (www.worldlifeexpectancy.com). The major causes of liver disease recorded in Burkina Faso are viral hepatitis infections of all types A, B, C, D, and E (Lingani et al., 2018; Tuaillon et al., 2018). Liver disease also can be induced to a lesser extent by liver parasites, i.e., plathelminth and helminth infections (Suzuki et al., 2011; Bagayan et al., 2016; Erismann et al., 2016); by chemical toxins; by mycotoxins (Warth et al., 2012); by alcohol consumption; and by tobacco smoking. Since 2002, Burkina Faso has been classified as an area of high HBV and HCV prevalence. HBV vaccination has been carried out since 2006 but is not completely protective (Sanou et al., 2018b). Moreover, there is very limited access to cancer treatment or pain medication. Therefore, of the 12.6% of the government budget allocated to public health, 60% is used for medical evacuation among which 80% are cancer patients.

For these reasons and according to ancestral practices, local populations are turning toward folk medicine for treatment with medicinal plants. A number of localized studies have reported on plants assumed to be hepatoprotective in Africa (Mukazayire et al., 2010; Kpodar et al., 2016; Muzumbukilwa et al., 2020), but there are no large-scale country-wide studies. In 1994, Burkina Faso adopted article 23/94/ADP into law, declaring traditional pharmacopeia as one component of the national health system. It recognized some traditional healers as part of the health care system and encouraged research into local medicinal plants. The government is aware of the benefit of medicinal research of local plants while complying with the conservation of biodiversity and of traditional knowledge. Our previous experimental work has shown that 1) traditional plant preparations and practices should be taken into account (Carraz et al., 2006; Ramdé-Tiendrébéogo et al., 2014; Sánchez-Carranza et al., 2017) and 2) because these practices can be healer-dependent (i.e., disease interpretation, plant material availability, and healer’s background), it was useful to perform a large-scale field study to collect sufficient data to allow us to look for repeated use of the same plants (Catarino et al., 2016; Henkin et al., 2017). We then decided in association with local traditional healers to carry out a full inventory of the Burkinabe plants used for hepatoprotective treatments. The record reported here represents a huge collection of healers’ knowledge of West African medicinal plants and of folk practices to heal liver disease.

## Materials and Methods

### Ethnopharmacological Survey

**FIGURE 1:**
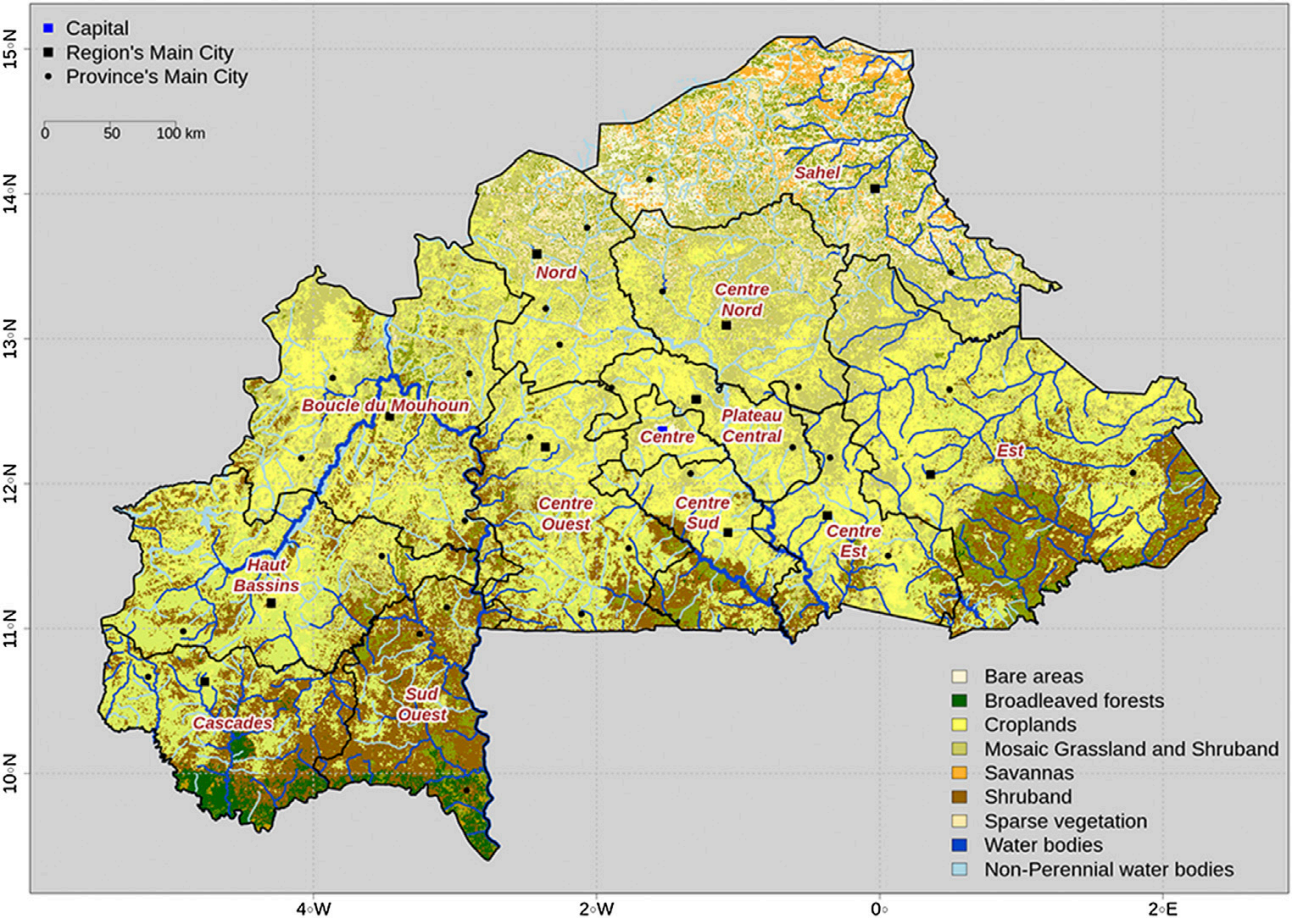
Geographic map of Burkina Faso with regional boundaries (in black), region names (in red), and main cities (blue and black dots).

Burkina Faso is a northwestern African country between latitudes 9°20′ and 15°5′N. It has a dry tropical climate with a monomodal rainfall distribution. The dry season lasts from November to April while the rainy season lasts from May to October and decreases in time and intensity from south to north (Ibrahim, 2012). Our survey took place from October 2013 to March 2014 and covered all 13 administrative regions of Burkina Faso (Figure 1). In each region, traditional healers had been contacted by the National Federation of Traditional Health Practitioners of Burkina Faso (FENATRAB) and asked for prior agreement. Therefore, the study included only practitioners who agreed to participate at this stage. Healers were selected for inclusion in our study if they specialized in liver pathologies and were said to cure them. Thanks to the FENATRAB, guides were identified in each region to assist the survey and translations if necessary. Consequently, surveys were carried out in the local language and translated into both French and English.

Data were recorded by nine investigators from the Department of Medicine and Traditional Pharmacopeia-Pharmacy (MEPHATRA-PH) of the Institute of Research in Health Science (IRSS) and from the University of Ouagadougou. The data collected included a long questionnaire with 31 main entries, as well as free discussions, field observations, and the geolocation of healers and plants. The questionnaire included five main sections: 1) information on the investigators, 2) information on the traditional healers, 3) the pathology treated and nosography, 4) information on plants, 5) plant harvesting, preparation, and administration of plants, and 6) information on plant toxicity.

### Identification of Plant Species

When possible, small plant samples were harvested for botanical identification at the Ecology and Vegetal Biology Laboratory of the University of Ouagadougou by the botanist Pr. Amado Ouedraogo. Voucher specimens were deposited at the herbarium of the University of Ouagadougou. Taxonomic designations of plants were checked online following The Plant List website (http://www.theplantlist.org). Sixty-nine plant entries (3.4%) could not be botanically identified because they are imported by healers from surrounding countries and no samples were available.

### Data Analysis

Data storage was performed on Sphinx Plus v5 software. R was used for all analysis, computation, and graphical representation, including maps and drawings. R is open source, distributed by the Comprehensive R Archive Network. Version 3.6.3 was used under a Linux Debian 10 host platform. The main libraries and associated dependencies used were 1) readr, dplyr, tidyverse, stringr, and splitstackshape for data cleaning and analysis, 2) raster and sp for graphs, and 3) maptools, geosphere, GADMTools, and geoname for maps.

Map layouts were generated based on compounds of available spatial datasets. Data sources are listed below:

1. Country and administrative boundaries dataset is from GADM (gadm.org), 2018
2. Land cover dataset is mainly from the Food and Agriculture Organization (FAO)/United Nations (fao.org), 2009, and from IGB-BDOT (Institut Géographique Burkinabé: Base de Données d’Occupation des Terres: igb.bf), 2002
3. Inland rivers and water areas datasets are mainly from the Digital Chart of the World (DCW 1992) with updates from the FAO dataset
4. Names of cities, populated places, and administrative regions were correlated with GeoNames (geonames.org) and data provided by the IGB

Finally, georeferenced data for healers and plants were linked to GNSS coordinates (Global Navigation Satellite System), acquired through the GPS (United States Global Positioning System) constellation, using eTrex Venture^®^ HC devices. Coordinates were serialized using N-S E-W designators over (decimal) floating point notation. Geodesics and distance between the healer and their plants were computed using Vincenty’s formulae; WGS84 ellipsoid parameters were used as inputs.

## Results

### Traditional Healers and Their Knowledge of Liver Disease in Burkina Faso

**FIGURE 2:**
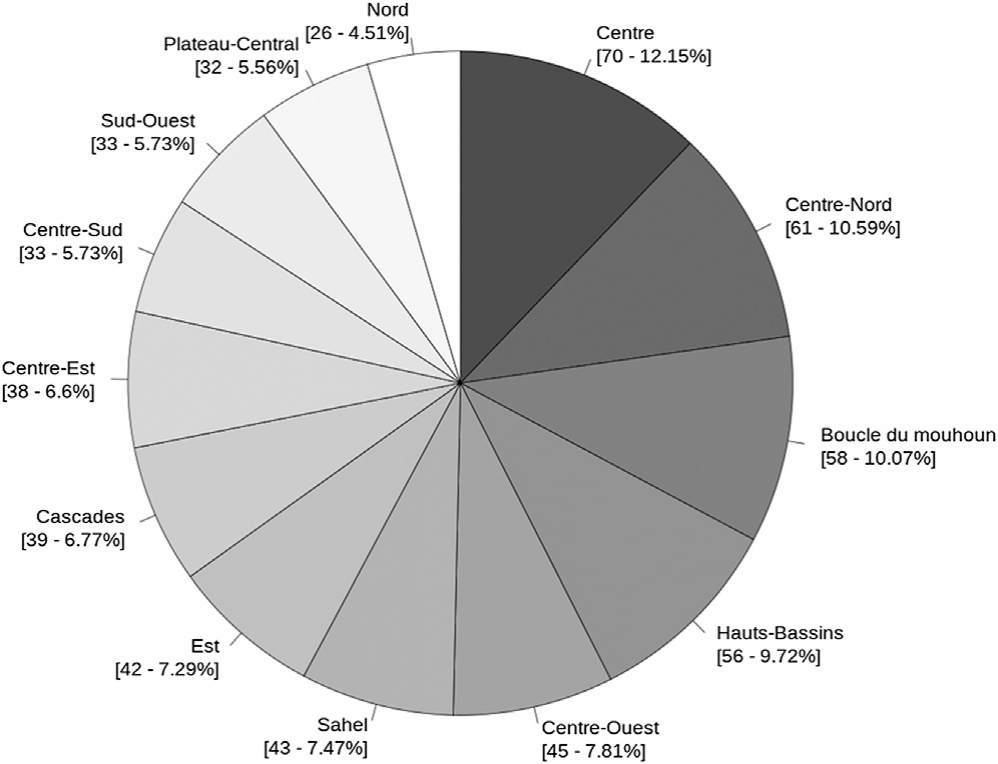
Distribution [records: %] of interviewed healers per region.

575 healers were interviewed throughout the country. They were evenly distributed across the 13 regions. Geographically, healers are more concentrated in the regions: Centre (12.2%, which includes the capital Ouagadougou), Centre-Nord (10%), Boucle du Mouhoun (10%), and Hauts-Bassins (10%) (Figure 2). As a result, 48.4% of the total traditional practitioners live in the central regions (Centre, Centre-Nord, Centre-Sud, Centre-Est, Centre-Ouest, and Plateau-Central), 32.3% in the west regions (Boucle du Mouhoun, Hauts-Bassins, Cascades, and Sud-Ouest), 12% in the north regions (Nord and Sahel), and 7.3% in the east region (Est). Among the 575 healers interviewed, 305 were women and 270 were male. Most healers were aged from 40 to 70 with a peak at age 50–60 (Supplementary Data S1). Only 15 healers wereunder 30 and 18 were over 80. During interviews, the healers described plants in at least 30 different languages and dialects. The main languages, spoken by 95% of the healers, are Mooré (44%), Dioula (21%), French (14%), Gourmantchéman (6%), Bissa (5%), Fulfuldé (3%), and Nouni (2%) (Supplementary Data S2). Moreover, even within the same dialect, some medicinal plants had different names or spellings.

**FIGURE 3:**
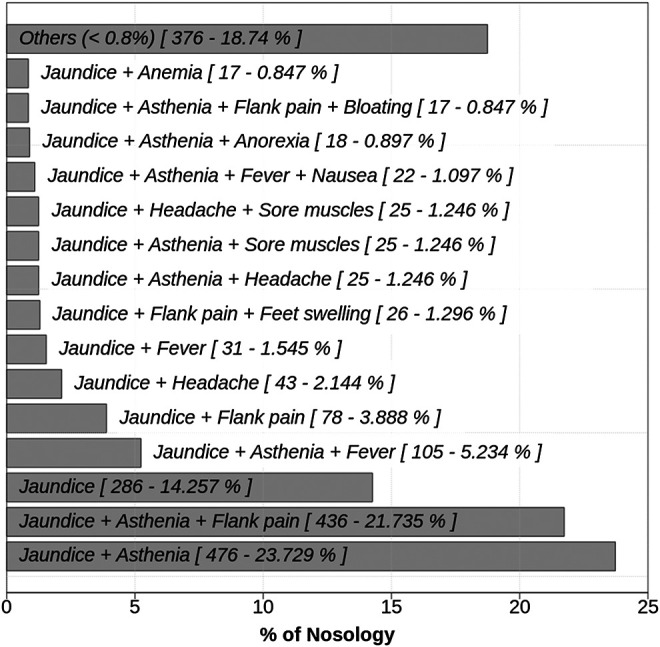
Nosology of liver disease in Burkina Faso according to healers.

All healers were familiar with liver diseases and described symptoms in 121 different combinations among which the most commonly described were asthenia and jaundice (23.7%), asthenia and jaundice and flank pain (21.7%), jaundice alone (14.3%), asthenia, jaundice, and fever (5.2%) (Figure 3). All symptoms were most commonly associated with jaundice. In addition, other symptoms were sporadically mentioned, some of which are related to liver pathologies (hepatomegaly, anorexia, nausea, headaches, and fever) and some are not (body aches, joint pain, cramps, feet and leg swelling, or itching) but the unrelated symptoms were quoted several times by different healers (Figure 3). Consistently, hepatomegaly, which needs auscultation, was reported 39 times by six different healers.

The liver pathologies that healers reported were in most cases jaundice (77.7%) which shows a possible confusion between a symptom and a pathology, followed by hepatitis (13.1%), unspecified liver dysfunction (5%), liver cancer (0.3%), cirrhosis (0.3%), or viral hepatitis (Table 1). Interestingly, we observed that each healer could specify separately one of these liver pathologies and give different plant remedies for each. Also, for each specific pathology, it was common for the healer to offer different solutions up to as many as 11 different herbal remedies for one condition. Also, some healers specified which type of the viral hepatitis they could treat: hepatitis B (2.6%), hepatitis A (0.5%), hepatitis A or B (0.2%), hepatitis B or C (0.15%), or hepatitis A, B, and C combined (0.1%). However, no healers reported a case of viral hepatitis C alone nor hepatitis D or E although these diagnoses are present in Burkina Faso. According to the healers, region Centre contained most cases of hepatitis (196 out of 230) and the three main viral hepatitis types: A, B, or C which are familiar for some Centre healers. Hepatitis B was mentioned in nine of the 13 regions but was not described in regions Centre-Ouest, Plateau Central, Nord, and Sud-Ouest. Hepatitis A was reported in regions Centre, Centre-Ouest, Boucle du Mouhoun, and Hauts-Bassins. Hepatitis C was only mentioned in regions Centre and Cascades (Supplementary Data S3).

**TABLE 1:** Liver pathologies in Burkina Faso according to healers.

Pathologies	% of citations
Jaundice	77.72
Hepatitis	13.11
Undetermined liver pathology	5.03
Viral hepatitis B	2.59
Viral hepatitis A	0.5
Cirrhosis	0.3
Liver cancer	0.3
Viral hepatitis A, B	0.2
Viral hepatitis B, C	0.15
Viral hepatitis A, B, C	0.1

## Selection and Collection of Medicinal Plants for Treatment of Liver Disease in Burkina Faso

**FIGURE 4:**
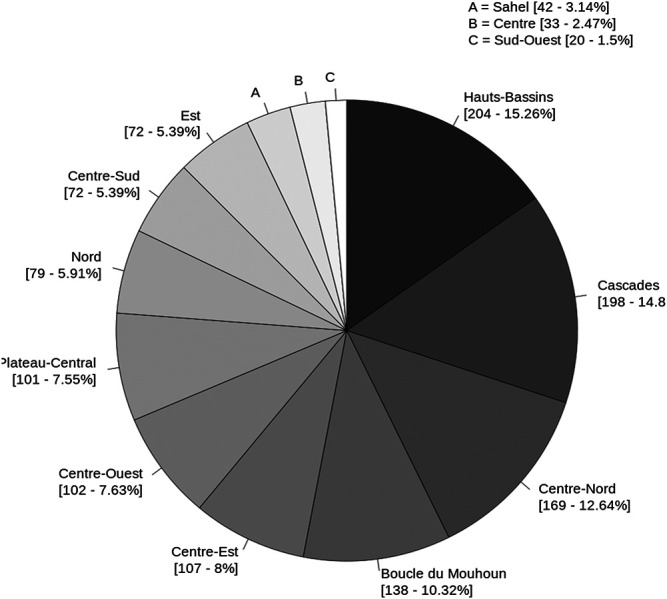
Distribution [records: %] of medicinal plants by region according their geolocation.

By exploring the 13 regions of Burkina Faso, 2,006 plant entries were collected. Analysis of their geolocation showed that these medicinal plants are actually distributed across the whole country but in a higher proportion in some regions such as Hauts-Bassins (15.3%), Cascades (14.8%), Centre-Nord (12.8%), and Boucle du Mouhoun (10.3%) (Figure 4). We then analyzed the number of plants prescribed by healers against liver disease and found an average of 3.3 plants per healer interviewed. When we analyzed the number of plants used by healers per region, it varied from 2.4 (in Sahel) to 4.4 (in Centre-Nord) (Supplementary Data S4). The number of plants recognized by healers ranged from one to 22. Only one healer mentioned 22 different plants to heal liver diseases and 107 healers (18.6%) mentioning using only one plant.

Botanical identification of the 2,006 plants resulted in 212 different plant species in 52 different plant families and 158 different genera, among which 127 are represented by only one species. The most represented families were Leguminosae (21.4%), Combretaceae (16%), Bixaceae (12%), Rubiaceae (5.5%), Meliaceae (4.4%), and Compositae (3.3%) (Supplementary Data S5). The genus cited most commonly by healers is *Cochlospermum* (12%), followed by *Terminalia* (6.4%), *Cassia* (4.9%). and *Anogeissus* (4.1%). The genera with the most diversified species are *Ficus* with seven different species, *Combretum* with six, and *Senna* with five. All 212 species were already reported to be African species. Among them, 60% are strictly or mostly African species, 22% are also found in other tropical countries (South America and Oceania), and 18% are more widely distributed worldwide. Finally, most plants were found in the wild. The nine most cited species were *Cochlospermum tinctorium* Perr. ex A. Rich. (Bixaceae) (186 healers; 9.3%), *Cassia sieberiana* DC. (Leguminosae) (88; 4.4%), *Anogeissus leiocarpa* (DC.) Guill. & Perr. (82; 4.1%), *Tamarindus indica* L. (Leguminosae) (73; 3.6%), *Terminalia avicennioides* Guill. & Perr. (Combretaceae) (64; 3.2%), *Carica papaya* L. (Caricaceae) (59; 2.9%), *Cochlospermum planchonii* Hook.f. ex Planch. (55; 2.7%), *Combretum micranthum* G.Don (55; 2.7%), and *Terminalia macroptera* Guill. & Perr. (55; 2.7%) (Table 2). Interestingly, among the most used plant species, two belong to the genus *Cochlospermum* and two others belong to the genus *Terminalia*. Domesticated plants were cited in lower numbers but they were widely used and included *Tamarindus indica* L., *Carica papaya* L., *Citrus limon* (L.), Osbeck (Rutaceae) (35 healers; 1.7%), and *Mangifera indica* L. (Anacardiaceae) (27; 1.4%), which can also be found widely distributed in other regions of the world. Different vernacular names are used for the plant species, depending on the local dialect. For example, *Cochlospermum tinctorium* Perr. ex A. Rich. is called sonsé or sonsé-yarna in Mooré; n’dribala or makou in Dioula; dafing, affeyana, or fayengu in Gourmantchéman; lougoure in Bissa; djaranbéré in Fulfuldé; tampoua in Nouni; and mimbon in Bwamu. The geolocation data for these most used species showed *Cochlospermum tinctorium* Perr. ex A. Rich which was found in six of the 13 regions while *Cochlospermum planchonii* Hook.f. in only one (Cascades). *Cassia sieberiana* DC. and *Tamarindus indica* L. were found in 10 regions, *Anogeissus leiocarpa* (DC.) Guill. & Perr. in 11 regions, *Terminalia avicennioides* Guill. & Perr. and *Carica papaya* L. in seven regions, *Combretum micranthum* G.Don in nine regions, and *Terminalia macroptera* Guill. & Perr. in three regions (Table 2; Supplementary Data S6).

**TABLE 2:** Selection of medicinal plants listed by healers against liver disease in Burkina Faso in descending order.

Voucher number	Plant names	Family	Record [%]	Most plant parts: record [%]	Number of regions	Used alone Yes/No: records	Records in combination	Most frequent associated species: records [%]
TIBIRI 4305 (OUA)	*Cochlospermum tinctorium* Perr. ex A. Rich	Bixaceae	186 [9.27]	R: 173 [93.01] Lf, R: 5 [2.69]	6	Y: 44	113	*Tamarindus indica*: 12 [8.45] *Terminalia avicennioides*: 11 [7.75]
TIBIRI 4374 (OUA)	*Cassia sieberiana* DC.	Leguminosae	88 [4.39]	R: 50 [56.82] Lf: 9 [10.23]	10	Y: 18	58	*Tamarindus indica*: 5 [7.14] *Xylopia aethiopica*: 3 [4.29]
TIBIRI 4373 (OUA)	*Anogeissus leiocarpa* (DC.) Guill. & Perr	Combretaceae	82 [4.09]	R: 50 [56.82] Lf: 9 [10.23]	11	Y: 16	59	*Cochlospermum planchonii*: 4 [6.06] *Terminalia macroptera*: 3 [4.55]
TIBIRI 4294 (OUA)	*Tamarindus indica* L	Leguminosae	73 [3.64]	Fr: 46 [63.01] Lf: 9 [12.33]	10	Y: 5	47	*Cochlospermum tinctorium*: 10 [14.71] *Cassia sieberiana*: 7 [10.30]
TIBIRI 4255 (OUA)	*Terminalia avicennioides* Guill. & Perr	Combretaceae	64 [3.19]	R: 23 [35.94] St: 21 [32.81]	7	Y: 15	35	*Cochlospermum tinctorium*: 11 [22.45] *Acanthospermum hispidum*: 2 [4.08]
TIBIRI 4316 (OUA)	*Carica papaya* L	Caricaceae	59 [2.94]	Lf: 49 [83.05] Lf, fr: 4 [6.78]	7	Y: 6	50	*Acanthospermum hispidum*: 2 [3.77] *Citrus limon* + *Senna occidentalis*: 2 [3.77]
TIBIRI 4419 (OUA)	*Cochlospermum planchonii* Hook.f.	Bixaceae	55 [2.74]	R: 53 [96.36] Lf: 1 [1.82]	1	Y: 19	30	*Anogeissus leiocarpa*: 2 [5.56] *Phyllanthus amarus*: 2 [5.56]
TIBIRI 4422 (OUA)	*Combretum micranthum* G.Don	Combretaceae	55 [2.74]	Lf: 42 [76.36] R: 6 [10.91]	9	Y: 17	32	*Chrysanthellum indicum*: 3 [7.90] *Cochlospermum tinctorium*: 3 [7.90]
TIBIRI 6836 (OUA)	*Terminalia macroptera* Guill. & Perr	Combretaceae	55 [2.74]	R: 40 [72.73] Lf, R: 5 [9.09]	3	Y: 9	41	*Anogeissus leiocarpa*: 3 [6.52] *Chrysanthellum indicum* + *Cochlospermum tinctorium* + *Isoberlinia doka*: 2 [4.35]
TIBIRI 4375 (OUA)	*Sarcocephalus latifolius* (Sm.) E.A.Bruce	Rubiaceae	46 [2.29]	R: 29 [63.04] Lf: 5 [10.87]	4	Y: 9	31	*Cochlospermum planchonii*: 3 [8.11] *Tamarindus indica*: 3 [8.11]
TIBIRI 4317 (OUA)	*Citrus limon* (L.) Burm.f	Rutaceae	35 [1.74]	Fr: 18 [51.43] Lf: 13 [37.14]	7	Y: 1	33	*Carica papaya* + *Senna occidentalis*: 2 [5.88] *Aframomum melegueta* + *Grewia lasiodiscus* + *Terminalia avicennioides* + *Xylopia aethiopica*: 1 [2.94]
TIBIRI 4280 (OUA)	*Guiera senegalensis* J.F.Gmel	Combretaceae	34 [1.69]	Lf: 24 [70.59] R: 4 [11.76]	10	Y: 2	29	*Combretum micranthum* + *Piliostigma reticulatum*: 3 [9.38] *Daniellia oliveri*: 2 [6.25]
TIBIRI 4383 (OUA)	*Mitragyna inermis* (Willd.) Kuntze	Rubiaceae	32 [1.6]	Lf: 19 [59.38] R: 7 [21.88]	7	Y: 1	31	*Adansonia digitata* + *Khaya senegalensis*+ *Tamarindus indica*: 1 [3.23] *Anogeissus leiocarpa* + *Cassia sieberiana* + *Cochlospermum tinctorium* + *Sarcocephalus latifolius* + *Stereospermum kunthianum*+ *Terminalia macroptera*: 1 [3.23]
TIBIRI 6837 (OUA)	*Trichilia emetica* Vahl.	Meliaceae	30 [1.5]	Lf: 13 [43.33] R: 12 [40]	4	Y: 4	25	*Tamarindus indica*: 2 [7.69] *Annona senegalensis* + *Bobgunnia madagascariensis* + *Cassia sieberiana*: 1 [3.85]
TIBIRI 4301 (OUA)	*Securidaca longipedunculata* Fresen.	Polygalaceae	29 [1.45]	R: 22 [75.86] Lf, R: 3 [10.34]	7	Y: 7	21	*Cochlospermum planchonii*: 2 [9.09] *Aframomum melegueta*: 1 [4.55]
TIBIRI 4309 (OUA)	*Chrysanthellum indicum* DC.	Compositae	27 [1.35]	WP: 16 [59.26] R: 7 [25.93]	2	Y: 9	16	*Combretum micranthum*: 3 [16.67] *Adansonia digitata* + *Afzelia africana* + *Combretum nigricans* + *Crossopteryx febrifuga* + *Eclipta alba*: 1 [5.56]
TIBIRI 4380 (OUA)	*Mangifera indica* L.	Anacardiaceae	27 [1.35]	Lf: 13 [48.15] St: 13 [48.15]	7	Y: 6	21	*Adansonia digitata* + *Carica papaya* + *Diospyros mespiliformis* + *Faidherbia albida* + *Ficus sycomorus* + *Piliostigma reticulatum* + *Sclerocarya birrea* + *Vitex doniana*: 1 [4.76] *Annona senegalensis* + *Vachellia nilotica*: 1 [4.76]
TIBIRI 4332 (OUA)	*Senna alata* (L.) Roxb.	Leguminosae	27 [1.35]	Lf: 12 [44.44] Fl: 4 [14.81]	6	Y: 14	12	*Cochlospermum planchonii*: 2 [15.39] *Acanthospermum hispidum* + *Aframomum melegueta* + *Combretum micranthum* + *Xylopia aethiopica*: 1 [7.69]
TIBIRI 4401 (OUA)	*Azadirachta indica* A. Juss	Meliaceae	25 [1.25]	Lf: 21 [84] St: 2 [8]	9	Y: 3	18	*Senna siamea*: 3 [13.64] *Eucalyptus camaldulensis*: 2 [9.09]
TIBIRI 4355 (OUA)	*Khaya senegalensis* (Desr.) A. Juss	Meliaceae	25 [1.25]	St: 17 [68] St, R: 2 [8]	6	Y: 2	20	*Azadirachta indica*: 2 [8.70] *Cassia italica* + *Tamarindus indica* + *Terminalia avicennioides*: 2 [8.70]
TIBIRI 4444 (OUA)	*Parkia biglobosa* (Jacq.) R.Br. ex G.Don	Leguminosae	25 [1.25]	St: 9 [36] Lf: 4 [16]	8	Y: 2	23	*Senegalia erythrocalyx* + *Anogeissus leiocarpa*: 1 [4.35] *Anogeissus leiocarpa* + *Mitragyna inermis*: 1 [4.35]
TIBIRI 4338 (OUA)	*Senna siamea* (Lam.) Irwin & Barneby	Leguminosae	23 [1.15]	Lf: 8 [34.78] Lf, Fl: 6 [26.09]	6	Y: 1	20	*Azadirachta indica*: 3 [13.64] *Acanthospermum hispidum* + *Senna alata*: 1 [4.55]
TIBIRI 4378 (OUA)	*Annona senegalensis* Pers.	Annonaceae	22 [1.1]	R: 15 [68.18] Lf: 4 [18.18]	8	N	21	*Cochlospermum tinctorium*: 2 [9.09] *Aframomum melegueta* + *Capsicum frutescens* + *Cochlospermum tinctorium* + *Combretum glutinosum* + *Terminalia macroptera* + *Xylopia aethiopica*: 1 [4.55]
TIBIRI 4425 (OUA)	*Eucalyptus camaldulensis* Dehnh.	Myrtaceae	22 [1.1]	Lf: 20 [90.91] Fr: 1 [4.55]	7	N	21	*Citrus limon* + *Senna siamea*: 2 [9.09] *Azadirachta indica* + *Carica papaya* + *Citrus limon*: 1 [4.55]
TIBIRI 4424 (OUA)	*Ximenia americana* L.	Olacaceae	22 [1.1]	R: 16 [72.73] Lf: 3 [13.64]	10	Y: 4	17	*Cassia sieberiana*: 2 [11.11] *Andropogon gayanus* + *Annona senegalensis* + *Anogeissus leiocarpa* + *Diospyros mespiliformis* + *Gymnosporia senegalensis* + *Lannea microcarpa* + *Sorghum bicolor*: 1 [5.56]
TIBIRI 4335 (OUA)	*Entada africana* Guill. & Perr.	Leguminosae	21 [1.05]	R: 10 [47.62] St: 6 [28.57]	4	Y: 6	14	*Faidherbia albida*: 2 [13.33] *Annona senegalensis* + *Combretum micranthum* + *Guiera senegalensis* + *Sarcocephalus latifolius* + *Terminalia macroptera*: 1 [6.67]
TIBIRI 4461 (OUA)	*Vernonia colorata* (Willd.) Drake	Compositae	21 [1.05]	Lf: 20 [95.24] Lf, R: 1 [4.76]	5	Y: 1	19	*Cochlospermum planchonii* + *Mangifera indica*: 2 [10] *Carica papaya* + *Citrus limon* + *Mitragyna inermis*: 1 [5]
TIBIRI 4329 (OUA)	*Senna occidentalis* (L.) Link	Leguminosae	19 [0.95]	Lf: 7 [36.84] Lf, R: 2 [10.53]	4	Y: 2	16	*Carica papaya* + *Citrus limon*: 2 [11.76] *Anogeissus leiocarpa* + *Cochlospermum tinctorium* + *Senna siamea*: 1 [5.88]

R, roots; Lf, leaves; St, stems; LSt, leafy stems; Fr, fruits; Fl, flowers; Se, seeds; WP, whole plant.

Regarding which plant parts were collected and used, roots and leaves are used the most, 32.9% and 28.2%, respectively, followed by the bark, leafy stems, and fruits (Supplementary Data S7). Seeds and flowers (1.3%) are not used much. The use of young leaves or young stems was sometimes specified as well as combinations of plant parts with leaves and roots being the most frequent (3.4%). In the case of *Cochlospermum tinctorium* Perr. ex A. Rich., all plant parts were used except the flowers, seeds, and fruits, with the roots being most commonly used (93%) (Table 2). In the case of *Cassia sieberiana* DC., all plant parts were used with roots being most commonly used (56.8%), while for *Anogeissus leiocarpa* (DC.) Guill. & Perr., the leaves were most commonly used (50%). Plant parts are harvested with knives or axes (47.2%) or with hands (27.9%) or both (24.8%).

**FIGURE 5:**
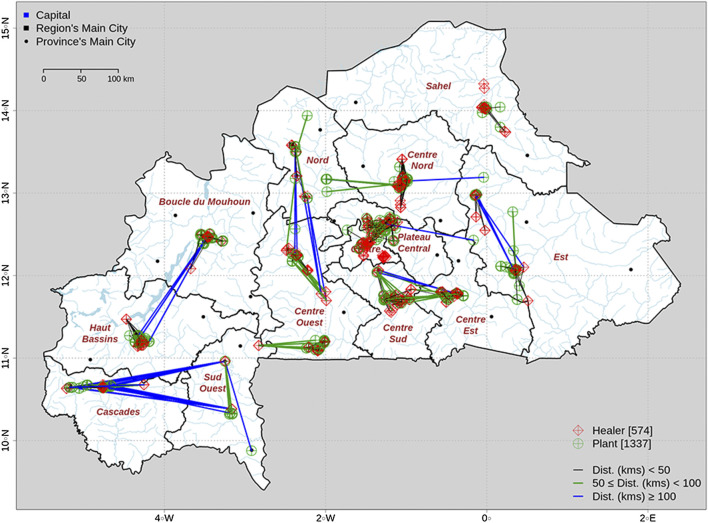
Map of interviewed healers (red dots), their plants (green dots), and distances between both according to geolocation.

We recorded the GPS coordinates of as many plants as possible and analyzed their location in relation to that of the healers’ home (Figure 5). Out of the 2,006 plant entries, GPS coordinates were not recorded for 668 entries (33%). For the 1,388 plants with GPS coordinates (66.7%), we strikingly revealed that some healers collect plant species very far away from their homes, traveling as far as the borders of their regions (Figure 5). The longest distances traveled (160–221 km) correspond mainly to healers from the region Sud-Ouest searching for medicinal plants in the Cascades. Such plants are *Anogeissus leiocarpa* (DC.) Guill. & Perr., *Cochlospermum planchonii* Hook.f., *Entada africana* Guill. & Perr., *Dichrostachys cinerea* (L.) Wight & Arn., *Cassia sieberiana* DC., and *Carica papaya* L. Healers may also travel very long distances within their region. For example, one healer in Sud-Ouest traveled 125 km to collect *Piliostigma thonningii* (Schumach.) Milne-Redh., and a healer in Est traveled 116 km to collect *Balanites aegyptiaca* (L.) Delile. In total, 97 plants were harvested from further than 100 km, 85 plants between 50 and 100 km, and 1,043 plants between 1 and 50 km (Supplementary Data S8).

In 7.7% of cases, plants were harvested at a specific time in their life cycle. For example, leaves of *Carica papaya* L. and *Combretum micranthum* G.Don were described as being collected before the plant bloomed. Some roots were collected specifically from aged specimens, for example, *Cochlospermum planchonii* Hook.f. ex Planch., *Cochlospermum tinctorium* Perr. ex A. Rich., *Pericopsis laxiflora* (Baker) Meeuwen, and *Zanthoxylum zanthoxyloides* (Lam.) Zepern. & Timler. Some roots could also be collected from dead plants such as *Gardenia erubescens* Stapf & Hutch. Blooming is obviously necessary when harvesting the flowers, but surprisingly some healers reported that blooming was necessary when harvesting the leaves or stem barks of *Anogeissus leiocarpa* (DC.) and the roots of *Chrysanthellum indicum* DC. According to 33 healers, plant parts should be harvested when the plant is fruiting, such as the leaves of *Anogeissus leiocarpa* (DC.) Guill. & Perr., *Senna occidentalis* (L.), *Terminalia macroptera* Guill. & Perr. and *Vitex chrysocarpa* Planch., or the roots of *Cassia sieberiana* DC., *Cissus populnea* Guill. & Perr., and tubers of *Cochlospermum tinctorium* Perr. ex A. Rich. Germination was also mentioned as being necessary for harvesting the leaves of *Anogeissus leiocarpa* (DC.) Guill. & Perr. or *Crotalaria goreensi* Guill. & Perr. Finally, some plants were harvested with spiritual practices (7.4% of cases). Spiritual practices included incantations to God or to forest spirit (5.1%) or creating a magic circle around the plant with ashes, salt, or sesame seeds (<1%). Several healers mentioned that the best time to harvest plants is in the morning or even before sunrise. One healer said that, for three of the seven plants they used, they should be collected on the patient’s birthday. Compelling testimonies were given where healers asked plants for forgiveness while harvesting them or harvested plants while naked as a sign of purity.

## Practices and Usages of Medicinal Plants for the Treatment of Liver Disease in Burkina Faso

During interviews, 139 plant species out of 212 (66%) were described as having other medicinal applications beyond treating liver disease. Again, we then searched for data consensus for each plant species (i.e., mentioned by at least three different healers), and we found the main secondary medicinal applications to be malaria (31.1%), chronic digestive illnesses including abdominal pain (9.1%), hemorrhoids (5.7%), and asthenia (4%) (Supplementary Data S9 and S10). Interestingly, the medicinal plants used to treat malaria were also most frequently used to treat liver disease. Conversely, 12 medicinal plants were described at least three times by healers without being associated with any pathology other than liver disease (Supplementary Data S10). Occasionally, serious illnesses were specifically mentioned by healers, such as HIV-AIDS, gout, diabetes, schistosomiasis, sickle cell disease, leishmaniosis, typhoid, ovarian cysts, and epilepsy. Regarding liver disease, four medicinal plants were reported to cure liver cancer, a fifth plant was reported to prevent liver cancer, and a sixth plant was reportedly able to fight any cancer. Moreover, these six plants were listed often (two to 29 times) by other healers against liver disease. Assumed anticancer medicinal plants came from four different healers. One healer in Ouagadougou described a recipe composed of a mixture of three plants: *Annona squamosa* L., *Vitellaria paradoxa* C.F. Gaerth., and *Senna siamea* (Lam.) H.S. Irwin & Barneby. Another healer in Ouagadougou, described the use of *Gardenia sokotensis* Hutch., possibly in association with six other medicinal plants. A third healer in Ouagadougou recommended the species *Lippia chevalieri* Moldenke which is reputedly preventative against liver cancer when patients have already contracted hepatitis. A fourth healer from Boucle du Mouhoun taught us that *Securidaca longipedunculata* Fresen. in association with *Calotropis procera* (Aiton) Dryand. could cure any cancerous wounds.

Most of the time, plant parts are harvested and used as either fresh or dry material (51.8%), or as specifically fresh material (30.9%) or as specifically dry material (17.3%). Plant preparations are mainly decoctions (69%), and then macerations are prepared in water or in local alcoholic beverages such as sorghum beers (8.2%) or powders of dried plants (4.6%) which are further mixed with soup, water, or millet porridge (Supplementary Data S11). Sometimes, some healers proposed different preparations for the same plant, depending on the pathology or plant combinations. Less often, plants are calcined or infused. The majority of recipes are then administrated orally (43.5%), or both orally and as a bath (39.4%). To a lesser extent, plants were also powdered and inhaled, used for massages, or used to evacuate the bowels.

Importantly, the listed medicinal plants are used, in most cases, in combination with two to 11 other plants (81.9%) (Table 3). Interestingly, those medicinal plants that are used most commonly in combination with other plants are the same that are used the most as the main remedy against liver disease. Only slight differences in their score ranks were recorded. Therefore, *Cochlospermum tinctorium* Perr. ex A. Rich. is used the most in various combinations (7.6%), followed by *Anogeissus leiocarpa* (DC.) Guill. & Perr. (4.1%) and *Cassia sieberiana* DC. (3.9%) (Table 3). Moreover, when listed as a co-ingredient, *Cochlospermum tinctorium* Perr. ex A. Rich. is usually associated with *Tamarindus indica* L. (1.3%) or with *Terminalia avicennioides* Guill. & Perr. (1.3%). Not all plants were used in combination; 363 plants, corresponding to 108 different plant species, were only used singly. Among them, 17 plant species were never mixed with any other species. *Cola cordifolia* (Cav.) R.Br. (Sterculiaceae) and *Elaeis guineensis* Jacq. (Arecaceae) were described independently twice without any associated plants (Supplementary Data S12). Besides plants, other ingredients are sometimes added to recipes. The most common are honey or potassium hydroxide. Instructions for treatment dosages range from teaspoonsful to a glassful of the plant remedy and are adapted to the age of the patient. In most cases, treatment with plant preparations was two (52.6%) or three (36.1%) times a day for several days depending on the remedy. Healers might recommend specific diets during plant treatments, such as fat-free, meat-free, alcohol-free, or salt-free meals.

**TABLE 3:** Most frequent medicinal plants listed in combination with other plants.

Plant names	Record [%]
*Cochlospermum tinctorium* Perr. ex A. Rich	417 [7.6]
*Anogeissus leiocarpa* (DC.) Guill. & Perr.	223 [4.1]
*Cassia sieberiana* DC.	212 [3.9]
*Tamarindus indica* L.	201 [3.7]
*Carica papaya* L.	187 [3.4]
*Terminalia macroptera* Guill. & Perr	166 [3.0]
*Terminalia avicennioides* Guill. & Perr	148 [2.7]
*Combretum micranthum* G.Don	141 [2.6]
*Sarcocephalus latifolius* (Sm.) E.A.Bruce	136 [2.5]
*Guiera senegalensis* J.F.Gmel	124 [2.3]
*Citrus limon* (L.) Burm.f	122 [2.2]
*Mitragyna inermis* (Willd.) Kuntze	108 [2.0]
*Cochlospermum planchonii* Hook.f.	106 [1.9]

Finally, plant remedies were assumed to be curative (57.8%) or were prescribed as both prophylactic and curative (41.8%). Treatment duration varied from 3 to 4 days (30.5% of cases), 1 week (30.8%), 1 month (6.4%), or until complete recovery (14%). Importantly, very few plant remedies (22 in total) were suspected to be toxic and 98.3% of plants used are declared non-toxic. Among those listed as toxic by at least two different healers, there were *Crinum zeylanicum* (L.) L. (Amaryllidaceae), *Bobgunnia madagascariensis* (Desv.) J. H. Kirkbr. & Wiersema (Leguminosae), *Anogeissus leiocarpa* (DC.) Guill. & Perr. (Combretaceae), *Sarcocephalus latifolius* (Sm.) E.A.Bruce (Rubiaceae), *Nicotiana tabacum* L. (Solanaceae), and *Dichrostachys cinerea* (L.) Wight & Arn. (Leguminoseae). However, all these medicinal plants were also listed as safe by other healers. Three more medicinal plants were quoted once in the survey but are known to be toxic. They were *Jatropha curcas* L. (Euphorbiaceae), *Lagenaria siceraria* (Molina) Standl. (Cucurbitaceae), and *Vitex chrysocarpa* Planch. (Lamiaceae). The combination of *Vitex chrysocarpa* Planch. (Lamiaceae) with *Jatropha curcas* L. was reported to possibly induce death. Healers have specified that toxicity in humans resulting from medicinal plants mainly impaired the brain, mouth, gut or esophagus, or fetal development.

## Discussion

We report results obtained from a large and unique ethnobotanical survey in Burkina Faso. Our goal was to get an overview of plants used to treat liver disease by a large number of healers across a whole country so we could analyze their use and hypothesize about which plants might exhibit a hepatoprotective effect. Such a study may differ from current ethnopharmacological surveys carried out in the social sciences but does not oppose them. We focused the study on liver disease because hepatotoxicity, viral hepatitis, and subsequently liver cancer have a high incidence in Burkina Faso and have become a public health issue. From the most commonly listed plants, we can expect a biological relevance that could justify their broader use as standardized phytochemicals against liver diseases. Moreover, by surveying healers on the one hand and plant uses and geolocations on the other hand, we participated in an update of both. In this study, 575 healers were interviewed. Although this number is very high compared to other ethnopharmacological surveys, it is still a small fraction of the estimated 30,000 healers in Burkina Faso (Zerbo et al., 2011). Women outnumbered men which differs from one previous field study (Tibiri et al., 2015). The knowledge embedded in African traditional medicine could be very different from Western knowledge in considering a disease in a more holistic way but also with a diagnosis based on symptoms which can have the weakness of sometimes not being specific (Lengani et al., 2010). Fortuitously, serious liver dysfunctions cause characteristic, physical symptoms such as yellowish skin and eyes, color changes in urine and stool samples, flank pain, and asthenia that Burkinabe healers clearly pointed out. Also, although concepts and etiology of cancer in traditional medicine are complex and are not always compatible with Western medical practices (Abubakar et al., 2007), liver cancer has been mentioned several times in this study. This might be due to the knowledge of high viral hepatitis prevalence in Burkina Faso, which causes liver cancer. Moreover, the fact that viral hepatitis A, B, or C is clearly cited by healers shows how traditional and conventional medicines are combined nowadays in tropical countries. However, no healer referred to HDV or HEV in our survey even though with 6.1% of prevalence, HDV infection in Burkina Faso is the highest in west Africa (Riou et al., 2016; Sanou et al., 2018a), and HEV prevalence is also high (19.1%) (Traoré et al., 2012; Traoré et al., 2016).

We have recorded a total of 2,006 plant entries against liver disease, corresponding to 212 different plant species, which represents around 10% of the total plant species of Burkina Faso (Thiombiano and Kampmann, 2010). This number confirms the vast knowledge of traditional medicine in the country (Nadembega et al., 2011; Megersa et al., 2013). Plants are mostly collected in the wild and harvesting of roots is a threat for biodiversity (Lulekal et al., 2008; Ugulu et al., 2009). This was a point of concern for the healers who we interviewed. The promotion of traditional medicine can result in ecological damage if no supportive measures are taken. One such measure might be the creation of botanical gardens of medicinal plants, with chemical and biological validation of remedies prepared from these plants. For now, when we analyze distances between healers and their plants by geolocation, we found some healers could search for a plant specimen very far away from their homes at distances of up to 200 km away. Considering the relevant plants are not rare, several explanations can be raised for this, such as habits of some healers transmitted by ancestral knowledge or by experimental observations that some species offer medical improvements when they grow in specific biotopes under the influence of particular atmospheric and soil conditions. On the same line, in 7.7% of cases, plant are harvested at specific stages of the plants’ life cycle, either before blooming, or when blooming (even when plant parts other than flowers are collected), when fruits are present, or from aged specimens. These observations may well have a biochemical relevance. The influence of soil and plant growth stages is currently being extensively studied (Jimoh et al., 2019; Moratalla-López et al., 2019; Peng et al., 2019). Also, the mode of plant preparations may influence the biological activity of medicinal plants (Carraz et al., 2006; De Donno et al., 2012). We believe that biochemical analysis techniques such as metabolomics could be very helpful to investigate the hypothesis that the active compounds in plant preparations might vary when collected at precise locations or specific plant life-stages or after a precise preparation mode. Another clarification of the study is that medicinal plants are mostly used as mixtures, prepared as decoctions and taken orally. This is consistent with previous studies of other pathologies (Asnake et al., 2016; Kantati et al., 2016; Ndob et al., 2016). Plant mixtures are justified by healers as either enhancing the therapeutic effect or minimizing the side effects of the main plant remedy. Therefore, we hypothesize that among the lead hepatoprotective plants reported here, some may have a direct curative effect on liver diseases (anti-viral, anti-parasitic, anti-inflammatory, anti-tumor, etc.) and some may have an indirect effect by detoxifying the liver after plants or mycotoxins or high glucose diet exposures. Commonly, both Burkinabe and Western people think that plants are non-toxic and do not cause unwanted side effects which is mistaken as shown by previous studies reporting allergies, kidney or liver failure, and death after plant treatments. Therefore, it appears remarkable in our study that some traditional healers recognized the possible toxicity of certain plant species such as *Crinum zeylanicum* (L.) L. (Amaryllidaceae), *Sarcocephalus latifolius* (Sm.) E.A.Bruce (Rubiaceae), *Jatropha curcas* L. (Euphorbiaceae), *Lagenaria siceraria* (Molina) Standl. (Cucurbitaceae), *Bobgunnia madagascariensis* (Desv.) J. H. Kirkbr. & Wiersema (Leguminosae), *Anogeissus leioc arpa* (DC.) Guill. & Perr. (Combretaceae), *Nicotiana tabacum* L. (Solanaceae), *Dichrostachys cinerea* (L.) Wight & Arn. (Leguminoseae), and *Vitex chrysocarpa* (Lamiaceae). Indeed, the toxicity of the first four of these species has already been proved experimentally (Candeias et al., 2009; Kuete et al., 2013; Shendge and Belemkar, 2019; Trebbi et al., 2019) on multidrug resistant cancer cell lines or rodent models.

According to the majority of Burkinabe healers interviewed in this study, the most useful plant species against liver disease are *Cochlospermum tinctorium* Perr. ex A. Rich. (Bixaceae), *Cassia sieberiana* DC. (Leguminosae), *Anogeissus leiocarpa* (DC.) Guill. & Perr. (Combretaceae), *Tamarindus indica* L. (Leguminosae), *Terminalia avicennioides* Guill. & Perr. (Combretaceae), *Carica papaya* L. (Caricaceae), *Combretum micranthum* G. Don (Combretaceae), *Terminalia macroptera* Guill. & Perr. (Combretaceae), and *Cochlospermum planchonii* Hook.f. ex Planch. (Bixaceae). Chemical extracts from the rhizomes of *Cochlospermum tinctorium* (concentration of 1 mg/ml) have been shown first to exhibit antihepatotoxic activity in primary cultured rat hepatocytes when cytotoxicity was induced using carbon tetrachloride (CCl_4_) or galactosamine (Diallo et al., 1987). Second, dose-dependent protection against CCL_4_ hepatotoxicity was shown in mice treated with an ethanol extract of *C. tinctorium* rhizomes as measured by alanine aminotransferase (ALT) levels in blood (maximal effect at 125 mg/kg of extract injected intraperitoneally) (Diallo et al., 1992). No toxicity assessment of such an extract had been carried out yet. *Cochlospermum planchonii*, known as *n’dribala*, is locally commercialized by the Phytofla laboratories for chronic hepatitis and malaria treatment. It was observed that in CCl_4_-treated rats, oral administration of an aqueous extract of the rhizomes prepared using traditional methods resulted in lowered serum levels of liver damage enzymes; this extract acts by inhibiting the cytochrome P-450 enzymes involved in the toxicity of CCL_4_ (Aliyu et al., 1995). However, this aqueous extract, when administrated orally to rats at 50 mg/kg over several days, showed some liver and kidney enzymatic toxicities which prevent its use (Nafiu et al., 2011). Several recent studies had been performed on *Tamarindus indica* extracts to prove their hepatoprotective activity and no toxicity has been detected in rabbits administrated orally with a high-dose ethanol extract of the leaves (Sinha et al., 2019). Studies have shown *T. indica* extracts can reduce the degree of hepatic steatosis in obesity-induced rats and can reverse the elevation of liver enzymes seen with a high-sugar high-fat diet (Azman et al., 2012). *T. indica* extracts were shown to effectively decrease liver damage biomarkers in further experimental models of liver intoxication, with either thioacetamide (Samal and Dangi, 2014) and CCl_4_ (Rodriguez Amado et al., 2016) in rats or dimethylhydrazine in hypercholesterolemic hamsters (Martinello et al., 2017). Limited chronic toxicity was detected when the *Carica papaya* extract was administrated at the therapeutic dose orally to rats (Afolabi et al., 2012) and rats intoxicated with arsenic but treated daily for three weeks with 150 mg/kg of an aqueous extract of the *C. papaya* root, exhibiting improvement in liver damage biomarkers and levels of antioxidant enzymes (Ojo et al., 2017; Sadek, 2012). In contrast, for the other commonly used species *Cassia sieberiana* DC., *Anogeissus leiocarpa* (DC.) Guill. & Perr, *Combretum micranthum* G. Don*, Terminalia avicennioides* Guill. & Perr., and *Terminalia macroptera* Guill. & Perr., no biological evaluation of their hepatoprotective properties has been carried out to date. These five species represent an interesting avenue for future laboratory investigations.

Six plant species were reported by four different healers to be helpful against liver cancer development. They were *Lippia chevalieri* Moldenke, *Annona squamosa* L., *Vitellaria paradoxa* C.F. Gaerth., *Senna siamea* (Lam.) H. S. Irwin & Barneby, *Gardenia sokotensis* Hutch., and *Securidaca longipedunculata* Fresen. So far, only extracts from the species *Annona squamosa* L. and *Vitellaria paradoxa* C.F. Gaerth. have been evaluated in liver cancer biological models. Thus, moderate antiproliferative activities (IC_50_ > 30 μg/ml) had been observed with methanol extracts of leaves, bark, and root of *V. paradoxa* on the human hepatocarcinoma cell line HepG2 (Mbaveng et al., 2011). Pericarp and seed extracts from *Annona squamosa* showed anti-hepatocarcinoma activity on HepG2 cells *in vitro* (IC_50_ of 0.36 μg/ml) as well as an anti-hepatoma activity *in vitro* and *in vivo* in transplanted tumor H(22) cells in mice (Chen et al., 2012; Chen et al., 2017). Moreover, although not mentioned by healers to be useful against liver cancer, anti-liver cancer properties were found with the fermented preparation of fruits of *Carica papaya* L. when hepatocarcinoma was induced by N-methyl-N-nitrosourea in mice (Somanah et al., 2016).

Burkinabe healers described other medical properties of plants beside their activity against liver disease; the most common being against malaria (31.1%). We have found that the plants used against malaria are also the plants most used to treat liver disease, which included *Cochlospermum tinctorium* Perr. ex A. Rich., *Cochlospermum planchonii* Hook.f. ex Planch., *Carica papaya* L., *Anogeissus leiocarpa* (DC.) Guill. & Perr., and *Cassia sieberiana* DC. Chemical extracts from these species have already been shown to inhibit the *Plasmodium* in experimental models of the blood stage (Ancolio et al., 2002; Sanon et al., 2003; Koudouvo et al., 2011; Ouattara et al., 2014; Abdulrazak et al., 2015; Lamien-Meda et al., 2015; Okpe et al., 2016). It may be the case that these plant species have a general mechanism of action on the liver as antiproliferative, antioxidative, or anti-inflammatory agents with a potent activity against *Plasmodium* at its hepatic stage (Mazier et al., 1985; Carraz et al., 2006); but none of these species had been tested on malaria as yet.

Finally, in the field, there is little feedback from patients who have been healed with traditional remedies; therefore, we plan to investigate patients to clarify which plant species have the greatest clinical relevance. Some cautions will apply because patient recovery might refer only to the relief of symptoms and may not represent a total clearance of viral hepatitis or liver cancer cells. Currently, the Burkinabe government is actively working to test local traditional medicine by proposing that traditional healers must demonstrate the therapeutic benefit of plant remedies on at least 30 patients before these remedies can be sold in pharmacies. We strongly believe that knowledge about medicinal plants should not only go from healers to patients and researchers but we also consider that researchers should inform healers of scientific discoveries made with these plants and provide training on pathology specificities. Sharing information between healers, patients, and researchers might increase the chance to find potent medicinal plants and improve national population health.

## Conclusion

This countrywide survey of plants used against liver disease was carried out with 575 traditional healers in all 13 regions of Burkina Faso. In total, 212 different plant species were reported with important details on the liver pathology targeted, geolocation of plants, preparation mode, and plant combinations. Data analysis of the resulting databank identified nine widely used plant species; the hepatoprotective activity of this group has been partially explored (four of the nine). Also, among six plant species reported as active against liver cancer, only two have been investigated to date. Therefore, this study paves the way for novel chemical and pharmacological assessments of plant extracts to validate plant-based traditional medicine and be of use to local and global patients.

## Data Availability Statement

The raw data supporting the conclusions of this manuscript will be made available by the authors, without undue reservation, to any qualified researcher.

## Ethics Statement

This study was approved by the Institutional Ethic Committee of IRSS (Burkina Faso), the Health Ministry of Burkina Faso and the written informed consent of the National Representative of Traditional Healers of Burkina Faso.

## Author Contributions

AT, AN, TT, SG, and NO performed the field study in Burkina Faso. AT, IG, and MC designed the project and the field study. J-MC and IO contributed to major clinical data. SB realized geolocation and statistical analysis and contributed to data analysis. AT and MC obtained the grants, analyzed and formatted the data, and prepared the text for publication.

## Funding

This work was supported by the BEST program from the Institut de Recherche pour le Développement (IRD) in France and the FONRID (Fonds National de la Recherche et de l’Innovation pour le Développement, grant session 2013) in Burkina Faso.

## Conflict of Interest

The authors declare that the research was conducted in the absence of any commercial or financial relationships that could be construed as a potential conflict of interest.
