# Supplementary material for: Countrywide Survey of Plants Used for Liver Disease Management by Traditional Healers in Burkina Faso
Source: Front Pharmacol. 2020 Nov 30;11:563751. doi: 10.3389/fphar.2020.563751 (PMC7883685; doi:10.3389/fphar.2020.563751)
Supplement: Supplementary file 1 [file datasheet1.zip › Supplementary data 1.docx]

**Supplementary data 1.** Healers’age distribution.
