# Supplementary material for: Countrywide Survey of Plants Used for Liver Disease Management by Traditional Healers in Burkina Faso
Source: Front Pharmacol. 2020 Nov 30;11:563751. doi: 10.3389/fphar.2020.563751 (PMC7883685; doi:10.3389/fphar.2020.563751)
Supplement: Supplementary file 1 [file datasheet1.zip › Supplementary data 4.docx]

**Supplementary data 4.** Average plants per healer ratio, by regions. Regions are those of healers, based on their geolocation.

| Regions | Number of different species | Ratio Plant/ Healer |
| --- | --- | --- |
| Centre | 225 | **3.2** |
| Centre-Nord | 270 | **4.4** |
| Centre-Ouest | 133 | **3.0** |
| Centre-Est | 150 | **4.1** |
| Centre-Sud | 110 | **3.3** |
| Plateau Central | 117 | **3.7** |
| Est | 128 | **3.0** |
| Nord | 68 | **2.6** |
| Sahel | 103 | **2.4** |
| Boucle du Mouhoun | 145 | **2.5** |
| Hauts-Bassins | 208 | **3.7** |
| Cascades | 128 | **3.3** |
| Sud-Ouest | 87 | **2.7** |
