# Supplementary material for: Countrywide Survey of Plants Used for Liver Disease Management by Traditional Healers in Burkina Faso
Source: Front Pharmacol. 2020 Nov 30;11:563751. doi: 10.3389/fphar.2020.563751 (PMC7883685; doi:10.3389/fphar.2020.563751)
Supplement: Supplementary file 1 [file datasheet1.zip › Supplementary data 8.docx]

**Supplementary data 8**. Distances between healers and plants according their geolocation.

| Distances (km) | Number of plant entries |
| --- | --- |
| 221 - 100 | 97 |
| 100 - 50 | 85 |
| 50 - 10 | 540 |
| 10 - 1 | 503 |
| < 1 | 112 |
| No GPS | 669 |
