# Supplementary material for: Countrywide Survey of Plants Used for Liver Disease Management by Traditional Healers in Burkina Faso
Source: Front Pharmacol. 2020 Nov 30;11:563751. doi: 10.3389/fphar.2020.563751 (PMC7883685; doi:10.3389/fphar.2020.563751)
Supplement: Supplementary file 1 [file datasheet1.zip › Questionnaire.docx]

Questionnaire for traditional healers on medicinal plants in Burkina Faso.

| **Investigator** | | |
| --- | --- | --- |
| Date: | Gender: | Home institution: |
| Identity code: | Function: |  |
| **Healer and location** | | |
| Geographical region: | Agreement to participate in the survey: | GPS coordinates: |
| Province: | Identity code: |  |
| Municipality: | Gender: |  |
| Town or village: | Age: |  |
| **Ethnomedical view on liver pathologies** | | |
| Pathology name: | Population affected: | Nosography: |
| **Herbal remedies identity** | | |
| Plant vernacular name: | Association with other plants? (Y/N): | Other pathologies healed by this plant: |
| Plant name language: | Plants associated: | GPS coordinates: |
| Botanical identification: | Association with other substances? (Y/N): |  |
| Plant family: | Substances associated: |  |
| **Harvesting method and herbal remedy preparation** | | |
| Plant parts used: | Harvest method (manual, knife, other): | Usage state of the plant (dry, fresh, both): |
| Phenology (flowering, germination, fruiting, other): | Harvest time (season, time of the day): | Preparation mode (decoction, macerate, infusion, carbonization, calcination, other) : |
|  | Free harvest description: | Free preparation description: |
| **Mode of administration and treatment by the herbal remedy** | | |
| Mode of administration (oral route, bath, inhalation, massage, other): | Frequency per day: | Treatment duration: |
| Free administration description: | Time of the day: | Treatment type (therapy/ prophylaxis, both): |
|  | Dosing per intake: |  |
| **Herbal remedy toxicity** | | |
| Toxicity (Y/N): | Adverse effects (Y/N): | Special precautions to be taken: |
| On which part of the organism the toxicity occurs: | Adverse effect description: |  |
| Free toxicity description: |  |  |
