# Supplementary material for: Countrywide Survey of Plants Used for Liver Disease Management by Traditional Healers in Burkina Faso
Source: Front Pharmacol. 2020 Nov 30;11:563751. doi: 10.3389/fphar.2020.563751 (PMC7883685; doi:10.3389/fphar.2020.563751)
Supplement: Supplementary file 1 [file datasheet1.zip › Supplementary data 9.docx]

**Supplementary data 9.** Other illnesses healed by the medicinal plant listed.

| Other illnesses | Records; % of citations |
| --- | --- |
| Malaria | 285; 31.1% |
| Bellyache | 83; 9.1% |
| Hemorrhoids | 52; 5.7% |
| Asthenia | 37; 4.0% |
| Swelling | 32; 3.5% |
| Diarrhea | 29; 3.2% |
| Constipation | 25; 2.7% |
| High blood pressure | 17; 1.9% |
| Sexual impotence | 13; 1.4% |
| Cough | 13; 1.4% |
| Gynecological pain | 12; 1.3 |
| Headaches | 10; 1.1% |
| Others (< 1%) | 112; 33.6% |
