# Supplementary material for: Countrywide Survey of Plants Used for Liver Disease Management by Traditional Healers in Burkina Faso
Source: Front Pharmacol. 2020 Nov 30;11:563751. doi: 10.3389/fphar.2020.563751 (PMC7883685; doi:10.3389/fphar.2020.563751)
Supplement: Supplementary file 1 [file datasheet1.zip › Frontiers_consent_form_CARRAZ.pdf]

frontiers

Consent Form

Title of Manuscript Countrywide scale survey of liver disease management by traditional healers in Burkina Faso

Manuscript ID 563751

Journal Frontiers in Pharmacology/Ethnopharmacology

Corresponding author Dr. Maëlle Carraz

I hereby provide consent for the publication of the manuscript detailed above, including any accompanying images or data contained within the manuscript that may directly or indirectly disclose my identity (or that of my child/ward/relative if signed by a parent or legal representative).
I understand that this information will be freely available online, and accessible to the general public. I understand that under the Frontiers terms of publication, this information may be reproduced and used for other purposes, including commercial uses. I acknowledge that this will reduce my actual privacy to the extent of the content of the manuscript.
I confirm that I have been given the opportunity to view the manuscript prior to publication, and I understand that once published, it cannot be removed from the published record except in exceptional circumstances.

Name

Signature

Date

If consent is being provided by a parent or legal representative, please complete the following:

Name of Representative COMPAORE Tibo Jean-Marie

Relationship of patient/participant President of National Traditional Healers (ANAPHARM. BURKINA

Signature [illegible]

Date 03/06/2020

NOTE: Please do not return this form to Frontiers. It should be completed and stored by the corresponding authors, in accordance with institutional policies. The form should be made available on request during review and post-publication.

Frontiers
Avenue du Tribunal-Fédéral 34 · 1005 Lausanne · Switzerland
Tel +41 21 510 17 00 · info@frontiersin.org · frontiersin.org
