# Supplementary material for: Countrywide Survey of Plants Used for Liver Disease Management by Traditional Healers in Burkina Faso
Source: Front Pharmacol. 2020 Nov 30;11:563751. doi: 10.3389/fphar.2020.563751 (PMC7883685; doi:10.3389/fphar.2020.563751)
Supplement: Supplementary file 1 [file datasheet1.zip › Supplementary data 12.docx]

**Supplementary data 12**. Medicinal plant species never used in combination with other plants.

| Plant names | Number of citation |
| --- | --- |
| *Cola cordifolia* (Cav.) R.Br. | 2 |
| *Elaeis guineensis* Jacq. | 2 |
| *Combretum aculeatum* Vent. | 1 |
| *Elionurus pobeguini* Stapf | 1 |
| *Excoecaria grahamii* Stapf. | 1 |
| *Fadogia agrestis* Schweinf. ex Hiern | 1 |
| *Ficus platyphylla* Delile | 1 |
| *Hibiscus physaloïdes* Guill. & Perr. | 1 |
| *Indigofera bracteolata* DC. | 1 |
| *Jatropha curcas* L. | 1 |
| *Loeseneriella africana* (Willd.) N.Hallé | 1 |
| *Maerua crassifolia* Forsk. | 1 |
| *Nymphaea lotus* L. | 1 |
| *Physalis lagascae* Roem. & Schult. | 1 |
| *Senna singueana* (Delile) Lock | 1 |
| *Solanum incanum* L. | 1 |
| *Vachellia tortilis* (Forssk.) Galasso & Banfi | 1 |
