# Supplementary material for: Countrywide Survey of Plants Used for Liver Disease Management by Traditional Healers in Burkina Faso
Source: Front Pharmacol. 2020 Nov 30;11:563751. doi: 10.3389/fphar.2020.563751 (PMC7883685; doi:10.3389/fphar.2020.563751)
Supplement: Supplementary file 1 [file datasheet1.zip › Supplementary data 3.docx]

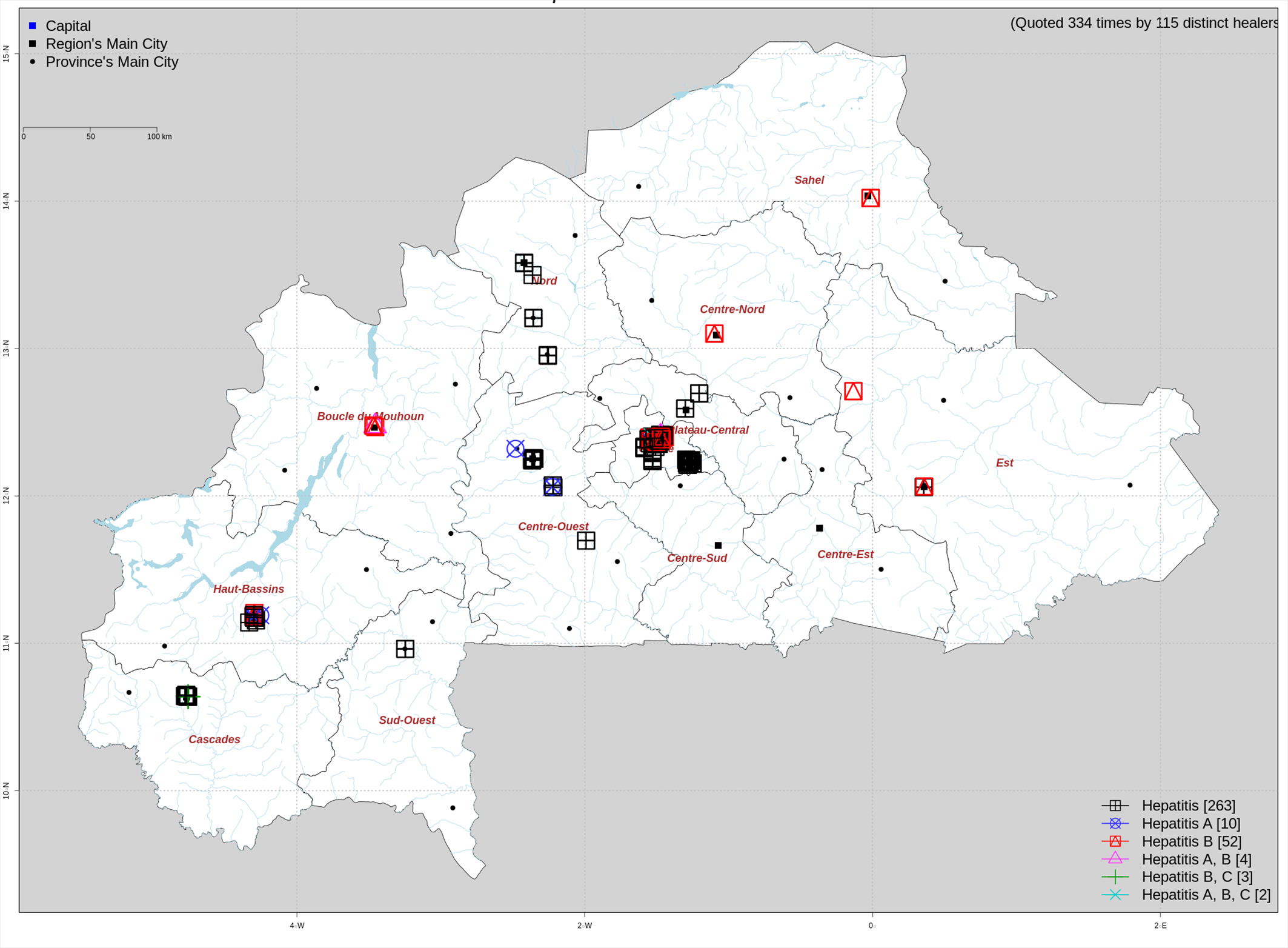


| **Hepatitis**  *Regions* | **ns** | **B** | **A** | **A, B** | **B, C** | **A, B, C** | **Healers’ quotes** |
| --- | --- | --- | --- | --- | --- | --- | --- |
| *Centre* | 196 | 27 | 2 | 3 |  | 2 | 230 |
| *Centre-Ouest* | 16 |  | 3 |  |  |  | 19 |
| *Centre-Nord* |  | 4 |  |  |  |  | 4 |
| *Plateau Central* | 4 |  |  |  |  |  | 4 |
| *Nord* | 14 |  |  |  |  |  | 14 |
| *Sahel* |  | 4 |  |  |  |  | 4 |
| *Est* | 3 | 5 |  |  |  |  | 8 |
| *Boucle du Mouhoun* |  | 3 |  | 1 |  |  | 4 |
| *Hauts-Bassins* | 12 | 9 | 5 |  |  |  | 26 |
| *Cascades* | 15 |  |  |  | 3 |  | 18 |
| *Sud-Ouest* | 3 |  |  |  |  |  | 3 |

**Supplementary data 3.** Distribution per region of healers’ assessment of hepatitis level. Ns: not specified.
