# Supplementary figures and images for: Countrywide Survey of Plants Used for Liver Disease Management by Traditional Healers in Burkina Faso
Source: Front Pharmacol. 2020 Nov 30;11:563751. doi: 10.3389/fphar.2020.563751 (PMC7883685; doi:10.3389/fphar.2020.563751)

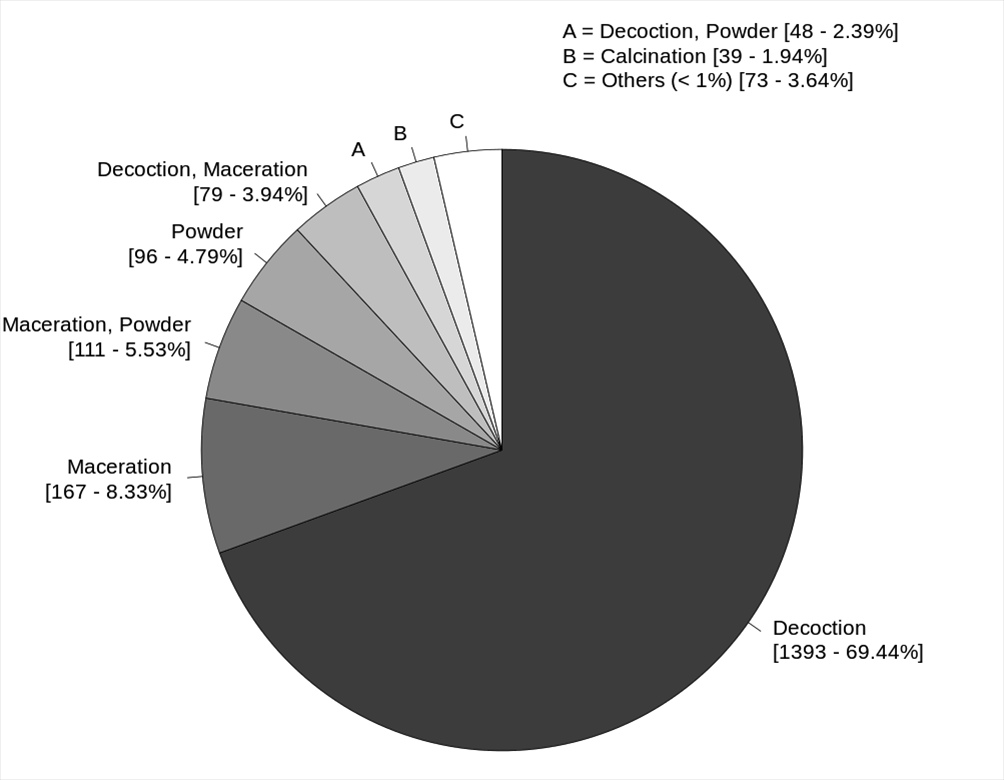


**Supplementary data 11**. Preparation methods of medicinal plants.

Supplement: Supplementary file 1 [file datasheet1.zip › Supplementary data 11.docx]

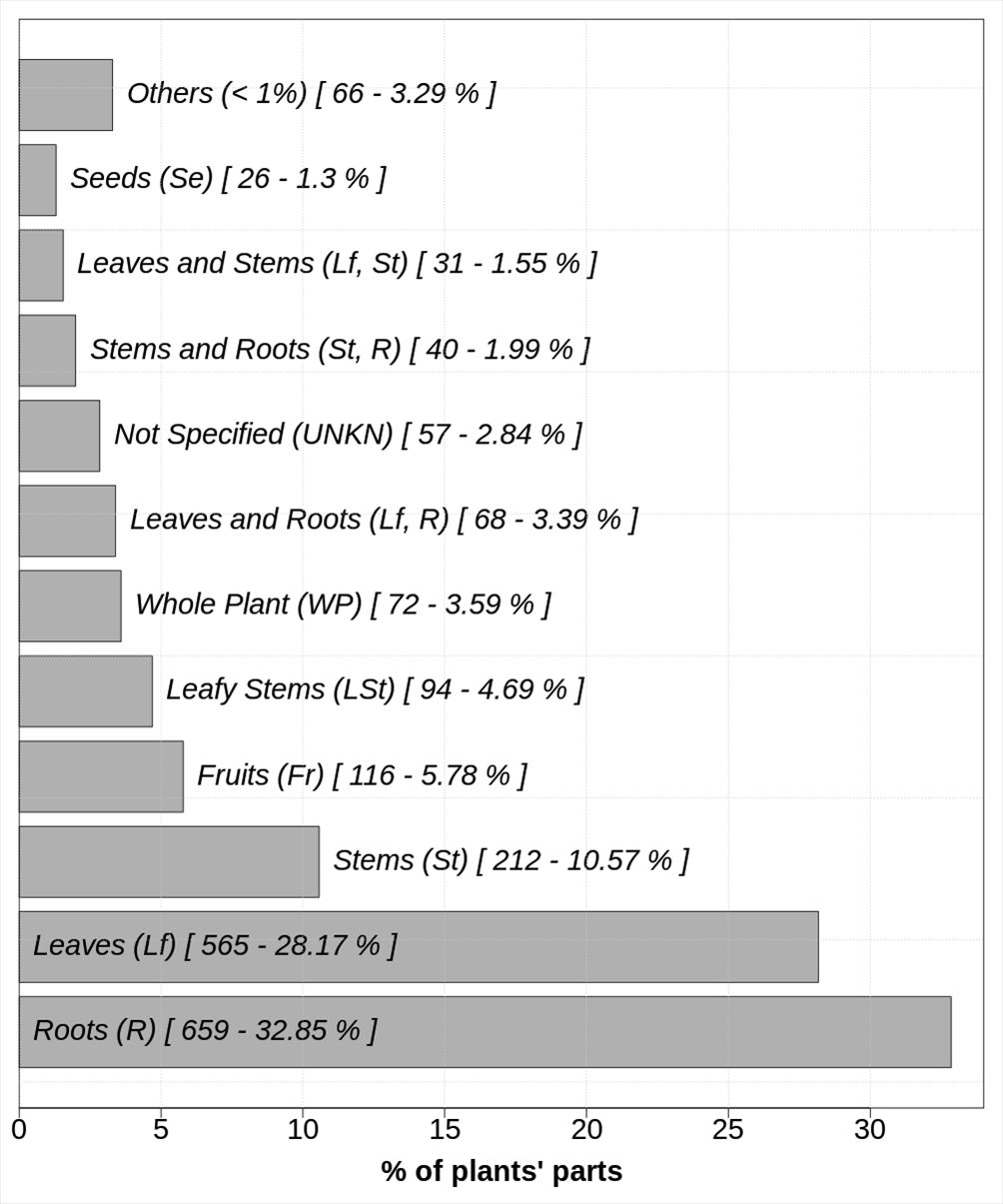


**Supplementary data 7**. Distribution [records - %] of medicinal plant parts used for remedies.

Supplement: Supplementary file 1 [file datasheet1.zip › Supplementary data 7.docx]

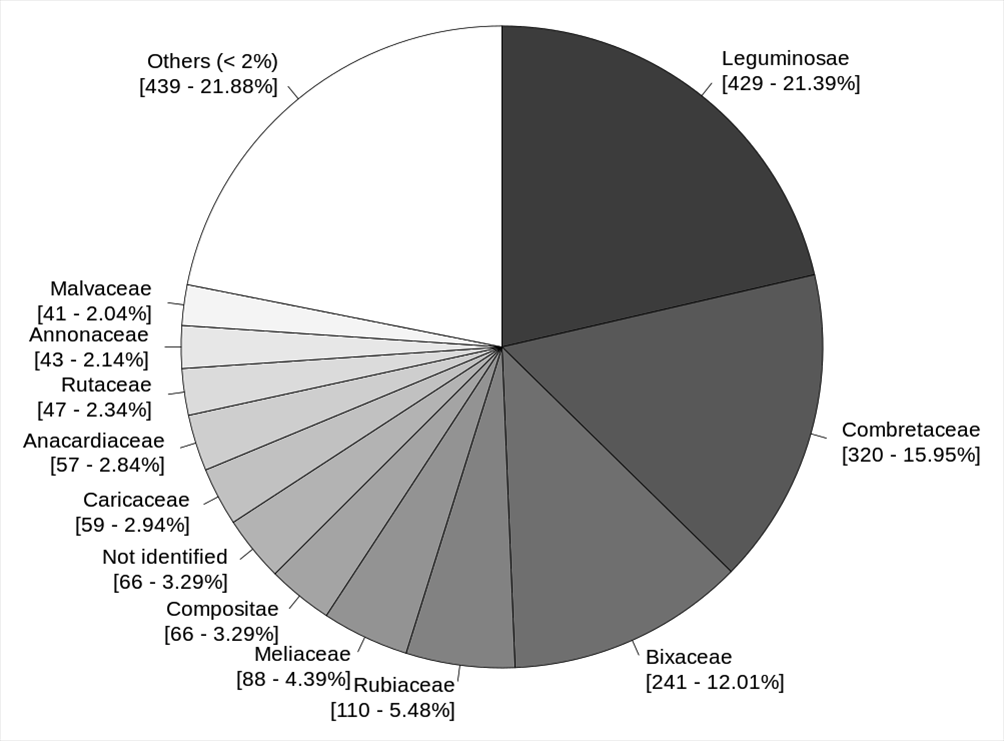


**Supplementary data 5**. Distribution [records - %] of medicinal plant families.

Supplement: Supplementary file 1 [file datasheet1.zip › Supplementary data 5.docx]

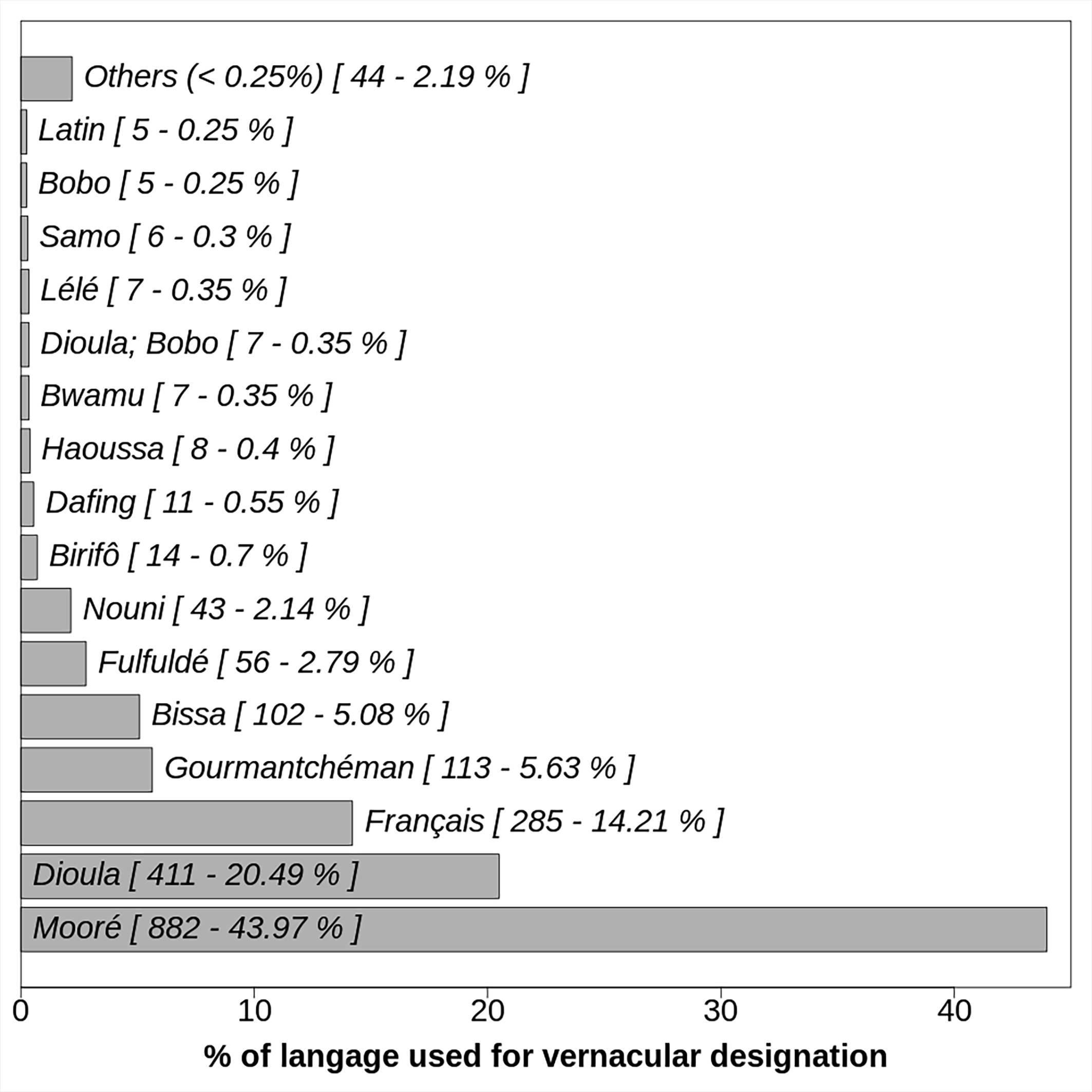


**Supplementary data 2.** Local languages used for plants’ vernacular names.

Supplement: Supplementary file 1 [file datasheet1.zip › Supplementary data 2.docx]
