# Supplementary material for: Countrywide Survey of Plants Used for Liver Disease Management by Traditional Healers in Burkina Faso
Source: Front Pharmacol. 2020 Nov 30;11:563751. doi: 10.3389/fphar.2020.563751 (PMC7883685; doi:10.3389/fphar.2020.563751)
Supplement: Supplementary file 1 [file datasheet1.zip › Supplementary data 6.docx]

**Supplementary data 6**. Geolocation maps of species *Cochlospermum tinctorium* Perr. ex A. Rich., *Cassia sieberiana DC.*, *Anogeissus leiocarpa* (DC.) Guill. & Perr., *Tamarindus indica* L., *Terminalia avicennioides* Guill. & Perr., *Carica papaya* L., *Combretum micranthum* G. Don, *Terminalia macroptera* Guill. & Perr., *Cochlospermum planchonii* Hook.f. ex Planch (Bixaceae) and distances to healers.
